# Academy of Medicine and Surgery, Richmond, Va.

**Published:** 1895-03

**Authors:** Mark W. Peyser


### RICHMOND (VA.) ACADEMY OF MEDICINE AND SURGERY.

FEBRUARY 12, 1895.

DR. LANDON B. EDWARDS was the leader in the subject for the evening: the therapeutics of gout, uric acid diathesis, gravel, etc.; the whole subject of gout, however, being open for discussion.

Poor man's gout is due to half-masticated food, washed down by draughts of heavy beer, and to lack of exercise. Gout may also be caused by lead. An excess of highly-seasoned food, in fact, excesses of any kind, directly predispose to it. The disease implicates, oftenest, the nervous system. Of course, it is directly due to the increased formation or diminished elimination of uric acid. There are strong reasons for attributing to the liver the chief part in the formation of urea. So long as this organ is active, the kidney carries it off; but if, for any reason, the kidneys and liver do not perform their duties, retention of uric acid occurs, and the results may be manifested in gout, regular or irregular, with its attendant symptoms, in the formation of tophi, or calculi—renal or vesical.

TREATMENT.—Children of gouty parents must do more than live temperately, both as regards food and drink. They must take plenty of exercise. An indication for treatment is to prevent catarrhal conditions of the urinary tract. Use milk and an abundance of alkaline waters. In the growing child, use fish, eggs, cereals, etc. Avoid overeating, especially highly-seasoned food and dark meats. Clothing must be suited to the season.

*Medicinal.*—In the examination of the urine, extractives should be looked for, as well as uric acid and albumen. For constipation, nothing is better than cascara. In the beginning of the attack, use moderate doses—twelve to fifteen drops—of the wine or tincture of colchicum seed, night and morning. Aconite, in drop doses, may be combined with it. The action of colchicum varies according to idiosyncrasy—some people being able to take a teaspoonful without discomfort, while small drop doses in others produce fatal effects; and, in any event, the vomiting, which may occur, is objectionable. Salicylate of sodium is a specific in this disease, and more. It assists in the elimination of uric acid, first, and then prevents its further formation. It shows its virtue in preventing both acute and chronic gout.

During the intervals, no one medicine excels iodide of potassium, in small doses, and at long intervals. For neurotic troubles, during the intervals, use dilute solutions of phosphate of sodium. Especial value is attributed to the use of the lithium salts. I have confidence in the lithia waters, especially for the results of gout. I have no doubt that calculi can be reduced in the system, and washed out by the urine. A case occurring in my practice is in point. The man claimed descent from noblemen. The father was a gourmand and beer-drinker, and his children had gout and renal calculus. In one child, the latter was pronounced. Operation was declined. The diet was restricted, Buffalo lithia water prescribed, and in a few days the urine was better, calculi passed, all symptoms disappeared, and, so far as I know, never reappeared. The effect of lithium and allied salts is to alkalinize the blood. It is proved by authorities that waters containing even but traces of lithium, possess solvent action in uric acid gravel and tophi. In one report, seventy-three per cent. of the cases collected, showed solution of stone, and in the balance crushing was facilitated.

### DISCUSSION.

DR. HUGH M. TAYLOR: Dr. Edwards said, in order to be free of gout, one must be poor. Now, according to authorities, the rich man has gout, but his child is free from stone; the poor man is free, but his child has stone. The reason is, the rich man's child has his food carefully selected, is given opportunities for taking all the exercise he wishes, and has no anxiety whatever. The poor man, although he eats indigestible, proteid food, and drinks heavy beer, is obliged to labor for his living. His child is scantily clothed, lives in unsanitary dwellings, and his food is not alone scanty, but indigestible. I have often been struck with the fact that vesical calculus is uncommon in the negro, and can recall but one case in the whole race in my practice. The explanation as to the adult is the same as in the case of the poor man, and I cannot understand why the child is exempt.

DR. JACOB MICHAUX: I am struck by Dr. Edwards' statement, as to the small quantities of salts acting favorably. If the doses are full, the stomach revolts, and especially is this true of the salts of lithium. We forget that we are injuring the digestive functions in giving large doses of alkali. The success of the mineral waters is due to the small amounts of the salts they contain, and to the fact that patients take them instead of water.

DR. J. S. WELLFORD: In the uric acid diathesis, there is not sufficient metabolism. I contend that rheumatism and gout are but different phases of the same thing. I don't believe in the lactic acid theory, and I hold that lithæmia is a blood-poisoning, due to uric acid, the same being true in the sequelæ of scarlet fever, and in dysmenorrhœa, most particularly in the latter, if the patient has gouty parents. In one case, in my practice, the patient was free during the reproductive period, but as soon as the menopause came on, gout manifested itself. Whenever a person is making more nitrogenous matter than he can dispose of, gouty symptoms occur. The skin plays an important part in elimination, as do the liver, intestines, etc. If, for any reason, they do not act, extra labor is thrown on the kidneys, and susceptibility to gout occurs.

My theory as to the cause of vascular and cardiac complications and sequelæ is that the lining membrane of the left side of the heart, and of the arteries is intended for alkaline fluid; that of the right side and veins, for acid. As soon as the blood, for any reason, becomes acid, the arteries and left side of the heart are affected, and we have arteritis, cardiac palpitation, angina and valvular trouble. We never have phlebitis in gout. As to the joints, they have less circulation than other parts of the body, and the blood is less alkaline. They are, therefore, disposed to stagnation and deposit.

Anything producing increased quantities of nitrogen or lessened metabolism, creates gout. After the food is taken in, digested, assimilated, it is rendered effete and thrown into the venous system. Prior to, and during this time, it undergoes chemical changes and leucomaines are formed. Urea is the first of these, then uric acid, and if indigestion occur, oxalic acid.

A number of people have gout because they do not take enough water, the uric acid being too concentrated.

In the *treatment* of gout, I am of the opinion that mischief may be done in trying to relieve too suddenly, by stopping elimination, and confirmed gout may result. Oil of peppermint is the best local application I have tried. It is soothing and aids in the solution of the acid. In constitutional treatment, don't use too much opium, for the reason given above; it prevents elimination of the acid. Of the alkalies, the salicylates, to a certain extent, are the best, especially that of potash. I believe the mineral waters subserve a useful purpose by flushing, and am satisfied that I have saved myself frequently from attacks of gout by the free ingestion of water.

Theoretically, lithium ought to be the best article in gout; but in my experience, I have not found it of half the value of potash the urate of the latter being more soluble. All of the potash salts are diuretic—soda, cholagogic. The main treatment should be in diet and exercise. The reason we have so much more gout now than previously, is to be found in the use of the street cars, and in the case of physicians, buggies. The diet must be regulated by the individual. No hard and fast lines can be set down. Let it be liberal. Diminish the nitrogenous food and liquors, or if the latter must be used, give good whisky. Use carbohydrates.

DR. MARK W. PEYSER referred to the connection between the leucocytoses and uric acid. The acid is related closely to the extractives zanthin, sarkin, gnanin, adenin, etc. Spleen nuclein does not contain uric acid nor these, but it does contain the mother-substance from which they may be made; the acid being formed in the presence of an oxidizing substance, the others being formed in its absence. The nuclein is derived from the colorless corpuscles and the amount of urea and uric acid formed is a measurer of nuclein metabolism. Any condition, then, which produces leucocytosis, increases the production of uric acid. Hence, we see it when a large amount of proteid food is taken in, in leucocythemia, phosporous poisoning, acute febrile diseases (especially pneumonia) in infants, pernicious anæmia, etc.

The doctor referred to the intimate relation existing between gout and diabetes mellitus and Packard in Hare's system of

Therapeutics quoted in regard to it. This authority cites table given by Charcot, showing that in three generations of one family, the first contained a gouty individual, the second, four gouty and four diabetic and the third, one gouty.

DR. E. C. LEVY mentioned a case illustrating the effect of diminished ingestion of liquids, which was speedily relieved by the free use of water.

The reason that carbohydrates are not advised, said the doctor, is because of the large amount of oxygen required for their oxidation. He has had good results in the use of piperazine.

DR. EDWARDS in closing the discussion, said that beginning with 1875, we could trace developing cases of gout. From 1860 to 1875, it was rare to hear of it, because of the struggle necessary for livelihood.

Piperazine has been tried and found wanting, clinically. The chemist in his laboratory found it perfect. Medical treatment should be tentative; drugs being given in small doses. Large doses of colchicum cause stomach troubles and are not inapt to produce death. "I am," he said, "in accord with Dr. Wellford as to relieving too suddenly." The salicylates act as solvents and not as the physiologists wished them to do. They are by far, the best remedies in the production of cure. As to diet, avoid tomatoes on account of the oxalic acid contained in them.

The presence of uric acid in the blood, greater than one to six thousand, will cause its precipitation and gout. One to seven thousand gives rise to the formation of crystals and the manifestation of gouty symptoms. Beyond this amount, it has no effect.

Dr. Wellford spoke of uric acid as a leucomaine. According to Roberts, hypodermic injection of it doesn't produce gout, and he (Roberts) says it is the mechanical action that gives rise to disease.

REPORTS OF CASES.

Dr. MICHAUX said he reported the following merely to go back to the subject for the coming discussion:

Male.—The first attack had been so sudden and severe that the patient thought he had been bitten by a spider; but subsequent attacks showed the nature of the malady. The diet was regulated, but the patient was imprudent. Finally, sali cylate of sodium with small doses of colchicum and aconite was administered, and the case progressed favorably.

"I am of the opinion," said the doctor, "that colchicum should be given in moderate doses short of its purgative action.

"Speaking of the injection of uric acid, I should hardly expect it to produce gout in the healthy individual, because the blood is alkaline and all the emunctories are in perfect action.

"In threatened attacks, I have employed free purgation with salines, with happy results.

---

FEBRUARY 26, 1895.

DR. HUGH McGUIRE read a paper on Lymphandenoma, commonly known as Elephantiasis; the former better expressing its pathology. The nature of the disease, its causes and symptoms were gone into and then the treatment was taken up.

If the disease is seen in its early stage, and proper treatment started, it may be relieved or, at least, held in check; but, if the trouble has become established, under our present methods little can be accomplished without the aid of the knife. During the inflammatory attacks the patient should be put to bed, hot or cold applications and the usual remedies for inflammatory troubles used. If the fever is high, anti-thermal agents should be employed. Tonics, such as quinine in cod-liver oil and the mineral acids can be profitably administered; but perhaps the most valuable medicament is iodide of potash. If the patient be living in a tropical country, he should, of course, be advised to move. After the inflammatory attack has subsided, inunction of iodine and mercurial ointment should be used to soften the skin and promote absorption. Much good has been done by firmly bandaging the part with a woolen, or better still, a rubber bandage. Lately, strong galvanic currents have been highly recommended. Ligation of the main artery of the limb and excision of a section of the sciatic nerve have been tried, and occasionally do good, but neither can be relied upon. In advanced cases of lymphandenoma of the genitals the affected parts should be amputated. Recent authorities also advise removal of large wedges of the affected tissue when the legs are involved. If the patient's condition does not allow removal of all the growth at one sitting, several operations may be done. In the operations the most rigid aseptic precautions are necessary, because of the intimate connection this growth has with the lymphatic system.

Before concluding, I would like to report an interesting case of lymphandenoma which has been under my treatment for some weeks.

L. C., colored female, aged twenty, has lymphandenoma of the lower limbs, the right more than the left. The greatest measurement of the right calf is 33 inches, thigh 35 inches. Both legs are eczematous. Seven years ago the patient suffered from ingrowing toe-nail of the right foot. An eruption started from this, the parts became hot and swollen, and she suffered from severe pain and fever for several days. In an average of once every one or two months she has had acute attacks of the disease, and after each, the leg has increased in bulk, until now it has reached an enormous size. About three years ago the left leg became involved and has steadily grown worse. The attacks are more severe in summer, and any unusual amount of work or walking will bring on the trouble, but complete rest of the limb is equally injurious, as then there is an accumulation of lymph in the part, causing great tension. Any abrasion of the skin is followed by a discharge of lymph, which gives temporary relief. During attacks she has sharp, shooting pains in the groin and calf, the limbs become stiff, glands swell and there is high fever. This condition lasts for a day or two, then gradually subsides, leaving the limbs larger and the general health impaired. Her legs are now enormous and locomotion is difficult.

In treating her, I have, at Dr. Hunter McGuire's suggestion, departed from the usual method. Three or four times weekly I apply over some of the main lymph channels of the leg a cup-shaped electrode which contains one day a saturated solution of iodide of potash, and the next, tincture of iodine. A galvanic current of seven or eight milliamperes is used for cataphoresis. Whether this treatment will give any permanent relief, I am, as yet, unable to say; but since it was begun the calf measurement has been reduced from 34 to 33 inches and the patient has passed a longer period without an acute attack than she has known for years. Her general health has been improved by tonics and she is advised to take a moderate amount of exercise.

The doctor exhibited photographs of the case.

DISCUSSION.

DR. HUGH M. TAYLOR: I am of the opinion that we do not know enough of the diseases of the lymphatic system. It has an important part in the economy closely related to that of the veins. The lymphatics are the great sewers; they cart away septic matter. To see how soon they act, watch a septic wound. But beside this, they convey antiseptic material and this should teach us the value of using antiseptics which are absorbed from the surface by the lymph radicles and carried to the deeper parts. As I understand the question, it is due to blocking of the deeper lymphatics, producing imflammatory trouble, overgrowth, etc., just as obstruction to the veins would cause œdema. Elephantiasis may start as a surface injury, spread through the lacunæ and radicles to the deeper vessels and main channels, and even affect the glands, but not necessarily the latter. The glands may be affected in disease without involvement of the vessels, acting as catch-pit for the septic material which has been conveyed to them by the pipes the lymph vessels. There are two forms of Elephantiasis: (1) Spurious, due to obstruction and inflammation of the radicles first and then the deeper channels; (2) True, due to a germ found in the tropics and semi-tropics. I can't see what treatment should be adopted, except to produce absorption of the obstruction.

DR EDWARD MCCARTHY thinks it would be difficult to get union in this disease when pieces are cut out as was stated by Dr. McGuire.

REPORTS OF CASES.

DR. V. W. HARRISON: I report this case because of the family history, which is novel to say the least. The patient had post-partum hemorrhage before the placenta was delivered. She was under chloroform, so the hand was introduced into the womb and the portion of the placenta not adherent, was peeled off with difficulty. She bled for twenty minutes, but made a recovery. Her two aunts died from post-partum hemorrhage. One was aged twenty-two and the other twentyfour years. Her mother had four children and after the birth of each there was hemorrhage. Two sisters, eighteen and nineteen years old respectively, died from post-partum hemorrhage.

Dr. Taylor: Male, aged forty, lawyer. Five years ago while pleading a case, the patient, who was a robust man, was taken with a sudden pain in the head, rendering him unable to go on. He was taken home and to a great extent lost his memory. Accompanying this there were no other troubles, as paralysis, etc. In this condition he remained for six weeks or two months; then he became better and was advised to give up practice and go to farming. He did this for two or three years and was doing well. About this time he had occasion to go to Baltimore. While on the street in that place, he suddenly lost the use of his lower extremities, but consciousness was retained. Control over the bladder and rectum was gone. He was sent to a hospital and in three or four months his bladder, rectum and locomotion had improved to some extent. In this condition he has been ever since. He walks as though he had locomotor ataxia; he has the girdle sensation and exaggerated reflexes; control over the bladder and rectum is only partial; constipation is present and there is a sense of numbness in the lower extremities. Mentally, he is whole and has returned to his vocation as lawyer.

I am not clear as to the cause of the trouble. When first taken (while he was laboring under mental pressure), I am of the opinion that some small vessel of the brain ruptured, producing effusion of blood, and consequent pressure. It was too sudden to be due to inflammation. In six weeks, the clot was absorbed, and there was restoration. The loss of locomotion and control over the bladder and rectum, were due to rupture of a vessel of the meninges of the cord, and not of one of the cord itself. I say it was hemorrhage, because it was sudden. Then, in six weeks or two months, this clot was absorbed, and the worse effects were partially, but not entirely, recovered from. I do not know if a better condition will result; if the disease does not progress, the prognosis is good. The only intelligent hypothesis I can give, is the rupture of the blood-vessels, due to an atheromatous condition. There is no history of specific trouble. For treatment, I am giving him fifty grains of iodide of potash a day.

DR. JOHN F. WOODWARD agrees that the trouble is hemorrhagic, and in the lumbar region. It is a mixture of locomotor ataxia and myelitis, the sensory and motor tracts of the cord being involved. "I am sure," said the doctor, "that one hundred and fifty grains of the iodide, instead of fifty, would give better results, as the following shows: A man seen at the eye, ear and throat clinic has lost the use of all the muscles of the eyeball and of the upper lid, the right eye being the one affected. The trouble was specific. When sixty grains a day had been reached, all motion, except that downward, was restored. The dose was increased to one hundred and twenty grains, and the eye moves perfectly."

DR. WM. S. GORDON: I do not agree with Dr. Woodward, that the case is one of true locomotor ataxia. If it is, some explanation is to be made. Of course, there was some predisposition in the brain to the attack. If the lesion was in the ascending or descending lateral tracts, the spinal symptoms would have been continuous with those of the brain, but the two are separate. If the case were a well-marked one of locomotor ataxia, we would have lightning pains. Besides a lesion of the posterior columns, the cerebellar tracts would be affected. Pressure, if light, would cause irritability and exaggeration of functions; if great, then abolition. I do not doubt that the cause of the disease is effusion.

DR. WOODWARD: Locomotor ataxia may begin months or years before its manifestation agreeing with the symptoms detailed by Dr. Taylor. There is an indication in the optic nerve long before, and also in the head, arms and legs. I did not say the case was one of true ataxia, but a mixture of it and myelitis. The fact of only a partial involvement of the sphincters proves it. It is hard to say to what the lesion is due. It is now contended that in locomotor ataxia, the seat of injury is in Spitzka's or Gower's column. Dr. Taylor's case may develop into one of true locomotor ataxia.

MARK W. PEYSER, Secretary.

